# A Review on the Current and Future State of Urinary Tract Infection Diagnostics

**DOI:** 10.3390/ijms262210847

**Published:** 2025-11-08

**Authors:** Łucja Dudzik, Paweł Krzyżek, Ewa Dworniczek

**Affiliations:** 1 The Provincial Specialist Hospital in Włocławek, 87-800 Włocławek, Poland; lucja.dudzik126@gmail.com; 2 Department of Microbiology, Faculty of Medicine, Wroclaw Medical University, 50-368 Wroclaw, Poland

**Keywords:** urinary tract infections, diagnostics, diagnostic techniques, uropathogens, antimicrobial resistance

## Abstract

Urinary tract infections (UTIs) constitute a severe global health problem, placing a significant burden on healthcare systems worldwide. These infections often lead to frequent hospitalizations, sick leaves, and serious post-infectious complications. One of the most critical aspects of UTI treatment is rapid and accurate identification of pathogens, which increasingly develop resistance to commonly used antibiotics, as well as to newer ones, despite the expectation of sustained efficacy. This alarming trend signals the widespread presence of strains equipped with multiple resistance factors. In this review, attention has been drawn to the current classification of UTIs based on age, gender, risk factors, and preferred treatment strategies. Additionally, this article reviews diagnostic solutions used in this matter, starting from those applied in routine diagnostics and ending with the latest, cutting-edge approaches that are gradually coming into use. Each method varies in its specificity, sensitivity, and costs of implementation, and therefore, the limitations associated with adopting these technologies in widespread UTI diagnostics are also discussed. The review emphasizes the need for further research to optimize these innovations and integrate them into broad clinical practice, ultimately enabling more effective combat against UTIs and limiting the spread of bacterial resistance.

## 1. Introduction

### 1.1. Prevalence

Urinary tract infections (UTIs) are among the most common bacterial infections worldwide. It is estimated that they affect 150 million people annually and can occur as either asymptomatic infections or infections with varying severity and localization [1]. While many cases are mild and resolve with appropriate treatment, undiagnosed or improperly managed infections can lead to serious complications, such as chronic infections, urosepsis, or kidney damage [2]. Epidemiological data from recent decades clearly indicate the growing problem of UTIs worldwide. A prime example is a study comparing the incidence of these infections between 1990 and 2019, which showed a 60% increase in the global number of cases [3]. Alarming statistics are emerging from various regions around the world. In the United States, UTIs were responsible for approximately 10.5 million outpatient visits, 3 million emergency department visits, and 400,000 hospitalizations annually. This scale of infection generated enormous costs for the health system, reaching $4.8 billion per year [4,5,6]. The situation in Europe is equally concerning [7,8,9]. Recurrent UTIs are one of the leading causes of work absenteeism and frequent medical consultations. For example, in France, between 2014 and 2018, a total of 2,083,973 patients were hospitalized with UTIs, resulting in an adjusted incidence rate of 900 cases per 100,000 inhabitants [7]. Similar observations apply to Poland. In a military hospital in Warsaw with 1000 beds, approximately 100 cases of urosepsis were recorded annually between 2022 and 2023, with an average hospital stay of 12.7 days. In a smaller hospital with 500 beds, the number of urosepsis cases averaged 50–60 per year [1]. Interestingly, a study comparing the incidence and management of UTIs in Germany and the United States revealed significant similarities. In both countries, patients with UTIs accounted for an average of 10% of physicians’ workloads, highlighting the scale of the problem regardless of geographic location [8]. This problem affects not only developed countries but also other regions worldwide. For example, in sub-Saharan African countries, the prevalence of UTIs between 2000 and 2021 was 32.12% [9]. In the UK, between 2018 and 2023, as many as 1.8 million hospital admissions were recorded related to UTIs [10]. In Norway, 82% of antibiotics are prescribed in primary care, and one in four prescriptions is issued for the treatment of UTIs [11]. In China, UTIs are the second most common type of infection [12]. In Japan, the prevalence among women over the age of 50 ranges from 6.0% to 8.6%, with higher rates observed in the older age groups [13].

These data clearly demonstrate that UTIs pose a significant challenge for healthcare systems worldwide, both from an epidemiological and economic perspective. The costs associated with hospitalization and treatment, as well as prolonged work absences, place a considerable burden on healthcare services. A large proportion of infections affect women of reproductive age [14]. It is estimated that approximately 11% of women experience a UTI each year [15], one in three women will have an uncomplicated UTI before the age of 24 [16], and the lifetime risk of developing a UTI in women exceeds 60% [17]. This is related to the unfavorable anatomical structure of the female urethra, which is significantly shorter than in men, and its close proximity to the perineal area facilitates the entry of bacteria into the bladder [18]. In women, it is also important to highlight pregnancy as a particularly critical period with an increased risk of UTIs. One of the key contributing factors is the rise in progesterone levels [19], which leads to the relaxation of smooth muscle throughout the urinary tract. Reduced ureteral tone and altered bladder dynamics promote urinary stasis, creating favorable conditions for bacterial growth and facilitating the ascent of bacteria from the urethra to the bladder and even the kidneys [20]. Additionally, the enlarging uterus can exert mechanical pressure on the ureters, obstructing urinary flow and further increasing the risk of UTIs in pregnant women [19]. UTIs in men are less common and are most often associated with functional or anatomical abnormalities [21]. Until recently, it was believed that any UTI in men should automatically be classified and treated as a complicated infection [22]. However, the new Hooton classification introduces the possibility of diagnosing uncomplicated UTIs in men [23]. After the age of 60, the incidence of UTIs in men increases, primarily due to prostate enlargement and urinary retention [24]. Nevertheless, even in this age group, women continue to account for the majority of UTI cases. UTIs in the elderly also represent a significant proportion of cases. It is estimated that asymptomatic bacteriuria occurs in up to 10% of men and 20% of women [25], and UTIs are responsible for 25% of hospitalizations in the geriatric population [26].

**Figure 1:**
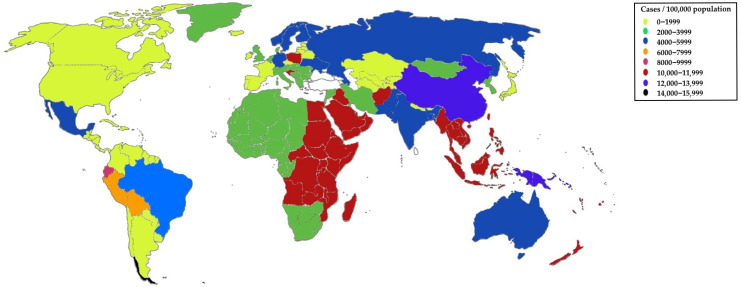
Age-standardized incidence data of urinary tract infections (UTIs) worldwide—epidemiological trends and predictions of UTIs from 2021 using the data of He et al. [27].

Based on the data of He et al. (Figure 1), the age-standardized incidence rate (ASIR) was presented worldwide [27]. ASIR indicates how many new cases of a given disease occurred per 100,000 people in the population, taking into account the age structure. The rate is standardized to a single, common reference population structure and includes both younger and older individuals. The highest ASIR was recorded in Ecuador—15,136.7 cases per 100,000 population. The lowest rate was observed in China—1184.13 cases per 100,000 population. Moreover, the most pronounced increase in ASIR for UTIs was observed in Mexico, while the greatest decrease was recorded in Italy.

UTIs present with a variety of clinical symptoms, but the most common include pain during urination, urgency, passing less urine, fever, and hematuria (Figure S1). From a clinical point of view, UTIs are divided into complicated and uncomplicated. Uncomplicated UTIs occur in individuals without anatomical abnormalities or risk factors (Table 1), whereas complicated UTIs are directly associated with such factors or caused by less frequent microorganisms, such as atypical bacteria or viruses [23,28].

### 1.2. Microbial Etiological Agents

The most commonly isolated microorganism responsible for UTIs is *Escherichia coli.* It accounts for as many as 75% of uncomplicated UTI cases and 65% of complicated ones. It is therefore the most common pathogen causing infections in both hospitalized and outpatient patients [29]. *E. coli*, as many other microorganisms, is capable of producing biofilm—a well-organized, three-dimensional structure of microorganisms surrounded by a polymeric substance. Biofilms exhibit special protective properties against external factors such as UV radiation, pH, or temperature. The matrix of the formed biofilm consists mainly of water. Other matrix components (polysaccharides, proteins, lipids and nucleic acids) provide their stability and support intercellular interactions. Biofilms play a key role in antibiotic resistance and tolerance. This is primarily due to the limited ability of drugs to penetrate the biofilm matrix. In addition, bacteria located in the deeper layers of the matrix exhibit reduced metabolic activity, which leads to decreased antibiotic effectiveness. The presence of specialized persister cells ensures that the biofilm survives unfavorable environmental conditions (e.g., during antibiotic therapy) and then rebuilds the damaged structure. All of these mechanisms contribute to making biofilms a serious clinical challenge [30]. Biofilm is formed by various microorganisms on tissues and implanted foreign bodies. Within the biofilm, frequent exchange of genetic material occurs between microorganisms, including genes responsible for tolerance, drug resistance, and the production of virulence factors. As a result, microorganisms may become more virulent, and the periodic transition from the sessile to the planktonic form can lead to a recurrence of acute infections.

Biofilms constitute a site for the accumulation of resistance genes and virulence factors, allowing microorganisms relatively easy opposition to antimicrobial therapies and intensification of host pathologies, respectively [31,32]. For example, uropathogenic *E. coli* (UPEC) strains can increase the synthesis of factors that enhance their invasiveness, such as alpha-hemolysin and cytotoxic necrotizing factor 1 (CNF1). Both of these factors are often associated with severe UTIs. Moreover, UPEC strains generally exhibit higher levels of antibiotic resistance compared to commensal *E. coli* strains, which complicates treatment and contributes to infection recurrence [23,29,33,34,35]. Another two important pathogens, *Klebsiella pneumoniae* and *Proteus mirabilis*, are strongly associated with UTIs. Due to their ability to acquire plasmids, these bacteria are characterized by frequent resistance to β-lactams. Additionally, their key feature is the ability to hydrolyze urea, which leads to urine alkalization and an increase in pH above 7.0. This process promotes the formation of urinary stones, which may serve as a reservoir for chronic infections [33]. Other species from the Enterobacterales order, such as *Providencia* and *Morganella*, are common causes of hospital-acquired infections, particularly in patients with risk factors [36,37]. Among non-fermenting Gram-negative rods, the most frequent cause of UTIs is *Pseudomonas aeruginosa*, which typically occurs in patients with hospital-acquired UTIs and those with indwelling catheters [29], while *Acinetobacter baumannii* is isolated much less frequently [38]. Among Gram-positive bacteria, the main causes of uncomplicated UTIs include *Staphylococcus saprophyticus*, a representative of coagulase-negative staphylococci, which is most often the etiological agent in young, sexually active women; *Enterococcus faecalis*, which is more common in patients with anatomical abnormalities of the urinary tract; and *Streptococcus agalactiae*, which is most frequently found in pregnant women or patients with diabetes [34,35]. *Staphylococcus aureus*, on the other hand, may act as an etiological agent in colonized patients or those undergoing urological procedures or catheterization [33,39]. In diabetic patients, there is also an increased risk of fungal UTIs. Although fungi are responsible for only 1–2% of all UTI cases, the most common etiological agent remains *Candida albicans* [33,40,41]. Other species from the *Candida* genus, such as *Candida glabrata*, *Candida parapsilosis*, *Candida tropicalis*, and *Candida kefir*, are much less frequently involved in such infections [42,43]. An increased risk of fungal infections is also observed in patients undergoing antibiotic therapy or immunosuppressive treatment [41]. They are typically etiological agents in complicated UTIs, with *C. albicans* being the most common. Adenoviruses, particularly type 11, are a frequent cause of hemorrhagic cystitis in children. In cases of sterile pyuria (>5 white blood cells per high-power field despite a negative urine culture), atypical bacteria may be the causative agents, including *Chlamydia trachomatis*, *Ureaplasma urealyticum*, *Mycoplasma hominis*, and *Mycoplasma genitalium* [36,37].

### 1.3. Treatment

Since the majority of UTIs are of bacterial etiology, antibiotic therapy remains the cornerstone of treatment (Table 2). In various parts of the world, first-line treatment for UTIs is similar. The most commonly used drugs include nitrofurantoin, sulfamethoxazole with trimethoprim, fosfomycin, and first-generation cephalosporins [28]. Outside the United States, pivmecillinam is also considered a first-line therapy [44]. Uncomplicated UTIs in women are most often treated with furazidin or fosfomycin. In men, antibiotics capable of penetrating the prostate gland, such as trimethoprim with sulfamethoxazole or fluoroquinolones, are recommended [27]. Despite their good tissue penetration, including the prostate gland, fluoroquinolones are recommended for the treatment of complicated UTIs, but should not be used for uncomplicated infections [45]. Studies have shown that resistance to fluoroquinolones can develop as early as three days after starting antibiotic therapy [46]. In the case of complicated infections, for example, in chronically catheterized patients, management depends on the patient’s clinical condition. A definitive diagnosis of catheter-associated urinary tract infections (CA-UTIs) requires bacteriuria in the range of ≥10^3^–≤10^5^ colony-forming units per milliliter (CFU/mL) along with a positive urinalysis (defined as a positive dipstick test, pyuria, or microorganisms seen in Gram-stained urine sediment) [47]. After CA-UTI is diagnosed, the catheter should be removed (if possible) or replaced with a new one before initiating antimicrobial therapy. Empiric antibiotic therapy may be broad-spectrum, but should later be optimized based on culture and susceptibility results [48,49]. The standard global approach consists of initial broad-spectrum empirical therapy followed by de-escalation to targeted therapy once microbiological results are available. Antibiotic selection should take into account local antimicrobial resistance patterns. Treatment of complicated UTIs typically lasts from 7 to 14 days [22], whereas in catheterized patients, a 7-day course appears effective in hospitalized patients with complicated UTIs, when agents with comparable intravenous and oral bioavailability are used; in other patients, treatment may need to be extended to 10 days [50].

To prevent CA-UTIs, it is recommended to limit the insertion and duration of catheters in patients, particularly those at higher risk of infection, such as the elderly, women, and immunosuppressed patients. Catheters should be used only when necessary, and after surgery, they should be removed as soon as possible (within 24 h). Adherence to hygiene and aseptic techniques, as well as staff/family education, also play a significant role [49]. Urinary catheters are widely manufactured by companies from many countries, including the United States (BD, Boston Scientific), Denmark (Coloplast), Germany (B. Braun), France (Vigmed), as well as China and India (numerous medical device manufacturers).

**Table 2:** Treatment of uncomplicated and complicated urinary tract infections (UTIs).

Type of UTI	Treatment	Comment [Reference]
Uncomplicated	Nitrofurantoin	Treatment of uncomplicated lower UTIs; effective against most Gram-positive and Gram-negative microorganisms [51]
	Sulfamethoxazole with trimethoprim	High rates of resistance preclude their use as empiric treatment of UTIs in several communities [52]
	Fosfomycin	High effectiveness against ESBL-producing strains [23]
	Furazidin	Higher antibacterial activity of furazidin compared to nitrofurantoin [53]
	Beta-lactams (amoxicillin with clavulanic acid, cefaclor)	It should be used for 7 days and its effectiveness is lower than that of fluoroquinolones [23]
	Fluoroquinolone	High effectiveness with 3-day treatment [23]
Complicated	Piperacillin-tazobactam	Treatment of *Enterococcus*, *Staphylococcus* or *Pseudomonas* [22]
	Piperacillin-tazobactam, fluoroquinolones, cefepime, or ceftazidime	Treatment of suspected *Pseudomonas* infections [23]
	Intravenous fosfomycin	Treatment of complicated UTIs, particularly ESBL-producing bacteria [23]
	Aminoglycosides	Reserved for patients in whom other antibiotics cannot be used due to resistance or allergy [19]
	Ceftazidime/avibactam	Considered a last-line antibiotic [54]
	Meropenem/vaborbactam	Effective against *K. pneumoniae* producing carbapenemases (KPC) [55]
	Plazomicin	Effective against the majority of *E. coli* and *K. pneumoniae* strains, including ESBL-producing strains [56]
	Tebipenem	Treatment of pyelonephritis and complicated UTIs [57]
	Cefiderocol	Synthetic siderophore cephalosporin; effective against many carbapenem-resistant bacteria [54]

Special attention should be given to multidrug-resistant strains and their resistance mechanisms, as they significantly limit the available therapeutic options for patients. Multidrug-resistant pathogens that pose a critical threat and require the development of new antibiotic classes include Enterobacterales producing cephalosporinases (e.g., extended-spectrum β-lactamases; ESBL) and carbapenemases, and *A. baumannii* and *P. aeruginosa* resistant to carbapenems. The second group includes *S. aureus* resistant to methicillin (MRSA) and vancomycin (VRSA), as well as *E. faecium* resistant to vancomycin (VRE) [58]. The primary resistance mechanism in *K. pneumoniae* and *E. coli*, the most commonly isolated enteric rods, is the production of ESBLs. These enzymes hydrolyze penicillins and most groups of cephalosporins, rendering the strains resistant to these antibiotics, although they remain susceptible to carbapenems. The production of ESBLs poses a significant threat due to the potential transfer of resistance genes between species via plasmids and transposons [59]. *K. pneumoniae* is the most common producer of carbapenemases and exhibits resistance to most, if not all, β-lactam antibiotics. Additionally, these strains are resistant to aminoglycosides, fluoroquinolones, and trimethoprim-sulfamethoxazole, leading to the development of an extensively drug-resistant (XDR) or pandrug-resistant (PDR) phenotype. In the case of methicillin-resistant *S. aureus* (MRSA), resistance is primarily associated with the *mecA* gene, which encodes modified penicillin-binding proteins (PBPs). As a result, these strains exhibit complete resistance to β-lactam antibiotics, with the exception of fifth-generation cephalosporins—ceftobiprole and ceftaroline. Hospital-associated MRSA strains demonstrate higher levels of antibiotic resistance. Due to the thickening of the peptidoglycan layer and cell wall, they have also developed resistance to glycopeptides. Enterococci, particularly *E. faecium* and *E. faecalis*, are increasingly significant pathogens in hospital-acquired infections [60]. In *E. faecium*, glycopeptide resistance is associated with the *vanA* and *vanB* genes, which are located on transposons, facilitating their spread [33]. Due to the naturally reduced susceptibility of enterococci to aminoglycosides, infections are typically treated with a combination of an aminoglycoside and either a penicillin or a glycopeptide. However, this approach is only effective if the strain does not exhibit acquired high-level aminoglycoside resistance (HLAR). Additionally, resistance to linezolid has been observed in *E. faecalis*, with daptomycin being a potential therapeutic option in such cases [60].

### 1.4. Aim of the Manuscript

As presented in the Introduction, the increasing antimicrobial resistance of uropathogens and the rising incidence of UTIs are problems of serious concern. Therefore, a rapid and precise identification of uropathogens is crucial for effective treatment. The implementation of modern diagnostic methods can significantly reduce the time required for pathogen identification, enabling faster initiation of effective treatment, preventing the spread of bacterial resistance, and reducing the risk of complications. In line with this, the aim of this review is to present the current and future diagnostic methods for UTIs worldwide. These methods will be categorized into three groups: commonly available methods, those used in higher-reference medical centers, and methods still in the research phase.

## 2. Methodology for Selecting Scientific Literature

Articles used to prepare the current review were searched with the Scopus and PUBMED databases. In that respect, only English-language and Polish-language articles were included. The applied terms were “diagnostics” or “diagnostic methods” together with “urinary tract infections” or “UTIs” or “uropathogens” or “urinary pathogens”. The selection of scientific literature sources was not limited to any specific time frames.

## 3. Common Diagnostic Techniques for UTIs

**Figure 2:**
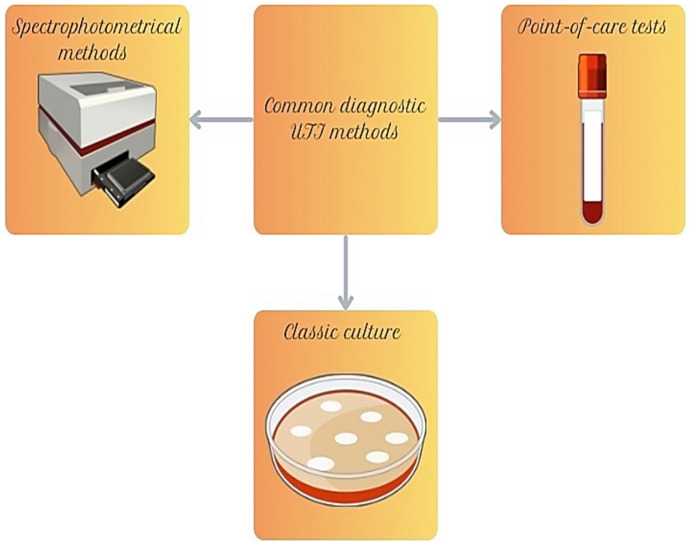
Laboratory methods commonly used in the diagnosis of urinary tract infections (UTIs).

Classical UTI diagnostic methods are cheap and allow for high-throughput microbial identification. These methods constitute a gold standard in first-line diagnosis of uropathogens and assist in distinguishing infection from colonization, especially in populations where symptoms may be unclear (Figure 2).

### 3.1. Culture and Disk Diffusion Tests

Diagnosis of UTIs has long been made based on the classical culture of microorganisms (Figure 2). However, over time, the media used have evolved, allowing for increasingly rapid and precise pathogen identification. In microbiological laboratories, two main groups of media are utilized: non-selective media, which support the growth of various microorganisms, and selective media, which restrict the growth of unwanted microorganisms and enable better diagnostics [61]. Among non-selective media, blood agar continues to play a key role, allowing the growth of both Gram-positive and Gram-negative bacteria and enabling the assessment of hemolytic ability. Although MacConkey agar is a selective-differential medium, due to the presence of bile salts and crystal violet, it selectively supports the growth of Gram-negative bacteria. CLED agar (Cystine-Lactose-Electrolyte-Deficient Agar) is one of the most commonly used selective media in UTI diagnostics—its lack of electrolytes prevents the swarming of *Proteus*, cystine supports the growth of coliforms, and the presence of lactose allows differentiation between fermenting and non-fermenting bacteria [62]. The most advanced media include chromogenic media, which enable rapid and intuitive bacterial identification based on colony coloration. One of the most commonly used is Brilliance UTI Clarity Agar, which, due to the presence of two chromogenic substrates, allows for the detection of bacteria such as *E. coli*, which grow as pink colonies, and *Enterococcus*, which form blue or turquoise colonies. Meanwhile, *Proteus*, *Morganella*, and *Providencia*, due to the presence of tryptophan, form colonies with characteristic brown halos. Another popular choice in UTI diagnostics is CHROMagar™, which, like other chromogenic media, facilitates the interpretation of results [63].

In the diagnosis of UTIs, it is essential not only to identify the type of microorganisms but also to determine their quantity. A quantitative urine culture is used to assess the number of microorganisms in a urine sample in order to distinguish UTIs from sample contamination. Urine cultures are performed quantitatively using a calibrated inoculating loop that collects 1 µL or 10 µL of urine. After colonies grow on the agar medium, the number of colony-forming units per milliliter (CFU/mL) is calculated by multiplying by ×1000 or ×100, respectively. Quantitative determination of microorganisms in urine samples is most commonly performed using a quantitative culture with a calibrated microbiological loop; however, in research settings, serial dilution methods, membrane filtration, or flow cytometry–based techniques may also be applied. Depending on the gender or type of infection, different thresholds are precisely indicated. Exceeding these values is interpreted as UTIs, whereas lower counts suggest the absence of UTI or sample contamination [28,64,65].

The cultivation of a bacterial strain on culture media allows for the determination of its antibiotic resistance. The disk diffusion method (Kirby-Bauer) involves measuring the zones of growth inhibition around antibiotic-impregnated disks to assess bacterial susceptibility [66]. Its main advantages include simplicity, low cost, and rapid result acquisition. However, minimum inhibitory concentration (MIC) values cannot be determined, and the test’s sensitivity depends on factors such as the type and thickness of the medium. To improve the reproducibility of results, strict guidelines have been introduced, including the use of Mueller-Hinton agar, standardization of bacterial suspension, precisely defined antibiotic concentrations in disks, and accurate methods for measuring inhibition zones [67,68].

Currently, additional variants of this method enable the detection of antibiotic resistance mechanisms (Table 3). One such test is the Double Disk Synergy Test (DDST), which is used to detect metallo-β-lactamases (MBLs). A disk containing a carbapenem (e.g., imipenem or ceftazidime) is placed on the plate along with a disk impregnated with EDTA, which chelates zinc ions—essential for MBL enzyme activity. The test evaluates the change in the size of the bacterial inhibition zone—if the initial zone around the antibiotic disk is small but significantly increases after the addition of EDTA, this suggests the presence of an MBL enzyme. Interpretation of results may be challenging in cases of highly resistant strains, a weak EDTA activity, or the presence of another carbapenemase, such as OXA-48. In such cases, additional tests are required to confirm the presence of these enzymes [69]. Temocillin disks are used as indicators to differentiate resistance mechanisms, as temocillin is resistant to hydrolysis by AmpC and ESBLs, but susceptible to hydrolysis by some other β-lactamases. For example, temocillin is hydrolyzed by class A β-lactamases such as TEM-1, SHV-1, OXA-1, as well as by OXA-48 carbapenemases. If a strain is resistant to both carbapenems and temocillin, this may suggest the presence of OXA-48 enzymes. In contrast, MBL-producing strains (e.g., NDM, VIM) often remain susceptible to temocillin while hydrolyzing carbapenems, allowing for a preliminary differentiation from OXA-48. At this stage, a hypothesis regarding the resistance mechanism can be formulated; however, definitive confirmation requires further studies, such as PCR-based molecular tests [70]. The CDT test (Combination Disk Test) is a disk diffusion method used to detect resistance mechanisms to carbapenems and AmpC β-lactamases. The procedure involves the use of two disks: one containing meropenem and the other meropenem combined with phenylboronic acid, which acts as an inhibitor of both carbapenemases and AmpC β-lactamases. After incubation, the difference in inhibition zones around the two disks is assessed—a larger inhibition zone around the disk with the inhibitor indicates the presence of an enzyme that degrades carbapenems or cephalosporins. However, due to the specific properties of phenylboronic acid, the test result requires additional verification to avoid misinterpretation [71]. CIM test (Carbapenem Inactivation Method) is not a standard disk diffusion test, as the antibiotic disk does not serve for diffusion in the agar medium but rather for assessing the inactivation of the antibiotic by the tested strain. The procedure involves incubating the bacteria in the presence of an imipenem disk, followed by transferring the suspension onto an agar plate inoculated with the standard control strain *E. coli* ATCC 25922, which is susceptible to carbapenems. If the tested strain produces carbapenemases, these enzymes break down imipenem, leading to its loss of activity. As a result, *E. coli* grows close to the disk, indicating the presence of carbapenemase production. Conversely, if a clear inhibition zone appears around the disk, it means that imipenem has retained its antibacterial properties and that the tested strain does not produce carbapenem-degrading enzymes. Thanks to this simple yet effective mechanism, *E. coli* ATCC 25922 acts as a biological indicator, enabling the detection of β-lactam-hydrolyzing enzymes in routine microbiological diagnostics [72].

Commercial disk-based tests for detecting various bacterial resistance mechanisms, including KPC, MBL, and OXA-48 carbapenemases, as well as ESBL, VRE, and MRSA, are available worldwide. Among the manufacturers offering such tests are MAST Diagnostica (Reinfeld, Germany), Rosco Diagnostica A/S (Taastrup, Denmark), and Liofilchem (Roseto degli Abruzzi, Italy). The presence of a carbapenemase in a tested strain is confirmed by a larger inhibition zone around the disk containing carbapenem and an inhibitor compared to the disk with carbapenem alone. Rosco and MAST commercial tests demonstrate nearly 100% sensitivity and high specificity (93%) in detecting KPC and NDM, although in some cases, they misidentified the simultaneous presence of both enzymes. The sensitivity for detecting VIM and IMP was significantly lower (50–58%) [73]. Other studies reported 86% sensitivity for MAST and Rosco tests, while Liofilchem achieved 96%, though it more frequently yielded false-positive results. The greatest difficulty was in detecting OXA-48 [73,74]. To detect ESBL, tests are based on comparing the activity of third-generation cephalosporins alone and in combination with a β-lactamase inhibitor—clavulanic acid. The presence of ESBL is confirmed by increased antibiotic effectiveness in the presence of the inhibitor [75]. For VRE detection, disks containing vancomycin are used, and in some tests, teicoplanin is also included to differentiate between VanA and VanB phenotypes [76]. MRSA is identified using cefoxitin-containing disks, as cefoxitin serves as a reliable marker for methicillin resistance [77].

**Table 3:** Disk diffusion tests are used to detect resistance to β-lactams.

Test	Disk Content	Type of Detected Antibiotic Resistance Mechanism
DDST	Disks with meropenem and EDTA [69]	Metallo-β-lactamases [69]
Temocillin disks	Temocillin [70]	TEM-1, SHV-1, OXA-1, OXA-48 [70]
CDT	Meropenem and meropenem combined with phenylboronic acid [71]	Carbapenems and AmpC β-lactamases [71]
CIM	Suspension onto an agar plate with *E. coli* ATCC 25922 [72]	Carbapenems [72]
MAST, Rosco, Liofilchem	One disk containing carbapenem and another an inhibitor compared with carbapenem alone [73]	KPC, MBL, and OXA-48 carbapenemases, ESBL and MRSA [73,77]

### 3.2. Point-of-Care Tests

Point-of-care tests are intended to provide rapid support in the diagnosis of UTIs (Figure 2). Currently, many such tests are available on the market; however, they differ in their mechanisms of action, sensitivity, and time to result. Most of them, although useful, still require improvement.

An example is the dipstick test by Siemens—Multistix 10 SG—which was used to analyze 635 urine samples from patients with positive urine cultures. The sensitivity of the dipstick test in detecting nitrites, leukocyte esterase, and blood was assessed. The results showed that nitrite and leukocyte esterase tests, when used individually, had low sensitivity and did not allow for reliable exclusion of UTIs. Therefore, the dipstick test alone is not sufficiently accurate as a standalone diagnostic tool. It is recommended that urine culture be routinely performed in such cases, regardless of the dipstick result. However, dipstick analysis may find application in outpatient settings and primary healthcare as a first-line screening tool. Researchers emphasize that test results should always be interpreted in the context of the patient’s clinical condition [78].

Flexicult^®^ is a diagnostic test that enables simultaneous quantitative detection of bacteria and assessment of their antibiotic resistance after approximately 18 h of incubation. The agar plate with a lid is divided into one large compartment for quantitative analysis and five smaller compartments containing antibiotics: trimethoprim, sulfamethizole, ampicillin, nitrofurantoin, and mecillinam. During testing, it was observed that the test more frequently indicated UTIs than laboratory culture. It was concluded that the test has a tendency to overestimate. Nevertheless, concordance in the identification of *E. coli* and assessment of antibiotic susceptibility was high [79]. In another study, it was found that the Flexicult test may help reduce unnecessary antibiotic prescribing, and its sensitivity and specificity were estimated as moderate (79% and 67%, respectively) [80].

Another test used in medical offices and classified as a point-of-care test is the Uricult Trio test, which is based on three agar media. In addition to CLED and MacConkey agars, it contains a selective medium for *E. coli*, intended for the detection of Gram-negative, β-glucuronidase-producing bacteria. A study involving pregnant women assessed the effectiveness of the Uricult Trio test. The sensitivity and specificity of the test were low, and *E. coli* was the most commonly detected pathogen, responsible for 36% of infections. It was concluded that the test is not sufficiently effective as a diagnostic tool for either asymptomatic or symptomatic bacteriuria [81]. Another study showed that, over the years, there has been no improvement in the performance of the Uricult Trio test. The results indicate that this test is only suitable for excluding UTIs or diagnosing UTIs caused by *E. coli* [82].

The DipStreak test is used for urine culture. Two agar surfaces are attached back-to-back on a plastic paddle. The device combines dip-slide technology with an original streaking mechanism, allowing for bacterial counting and colony isolation. In one study, DipStreak achieved a detection rate of 99.7%, and in another study, 99.4%, which is comparable to the results of standard laboratory methods. The sensitivity for the presumptive identification of pathogens such as *E. coli*, *P. mirabilis*, and *Enterococcus* was 97% and 88% in the respective studies. Additionally, it is noted that DipStreak allows for bedside inoculation and safe transport of the sample to the laboratory, which reduces the risk of bacterial overgrowth and lowers the number of false-positive results [83].

### 3.3. Automated Spectrophotometric Systems

Many diagnostic laboratories are also frequently equipped with specialized spectrophotometric systems (Figure 2). One of the most commonly used systems for determining bacterial susceptibility is VITEK 2, developed by bioMérieux, Marcy l’Etoile, France. This device operates based on spectrophotometry, analyzing bacterial growth in the presence of antibiotics using specialized test cards. Thanks to this technology, MIC values can be determined quickly, and resistance mechanisms such as ESBL, KPC, MBL, OXA-48, MRSA, and VRE can be detected. Compared to manual methods, VITEK 2 significantly accelerates diagnostics, reducing the waiting time for results to 4–8 h [84]. In addition to VITEK 2, other automated systems are also found in laboratories, such as the Phoenix system (BD), a diagnostic system used for bacterial identification and antimicrobial susceptibility testing. It employs two main measurement techniques: fluorescence analysis and turbidimetry. Fluorescence detects bacterial metabolism through enzyme activity, which emits light in response to specific substrates. Turbidimetry, on the other hand, measures the degree of medium turbidity, assessing how intensely bacteria proliferate in the presence of antibiotics. Using these methods, BD Phoenix precisely determines the MIC and detects antibiotic resistance mechanisms such as ESBL, KPC, and MRSA. The BD Phoenix system identifies ESBL by analyzing bacterial growth in the presence of third- and fourth-generation cephalosporins and a confirmatory test with clavulanic acid—if the MIC decreases after adding the inhibitor, it indicates ESBL production [85]. MRSA is identified based on a high MIC for oxacillin or cefoxitin, which suggests the presence of the PBP2a protein, being responsible for β-lactam resistance [77]. KPC detection is based on a high MIC for carbapenems [86]. Other systems include MicroScan (Beckman Coulter, Inc. Atlanta, Georgia, USA) and Sensititre (Thermo Fisher, Waltham, MA, USA), both of which use the broth microdilution method for determining bacterial susceptibility. In both cases, bacteria are inoculated onto microplates containing different concentrations of antibiotics and then incubated while the system monitors their growth. MicroScan analyzes medium turbidity (turbidimetry), allowing for precise determination of the MIC, particularly for rare resistance mechanisms. On the other hand, Sensititre offers the advantage of testing the latest antibiotics, including those used against multidrug-resistant strains [87]. Both systems provide high diagnostic accuracy, though they require more time than fully automated methods such as VITEK 2 or BD Phoenix, with results available after 8–12 h.

## 4. Specialistic Diagnostic Methods for UTIs

**Figure 3:**
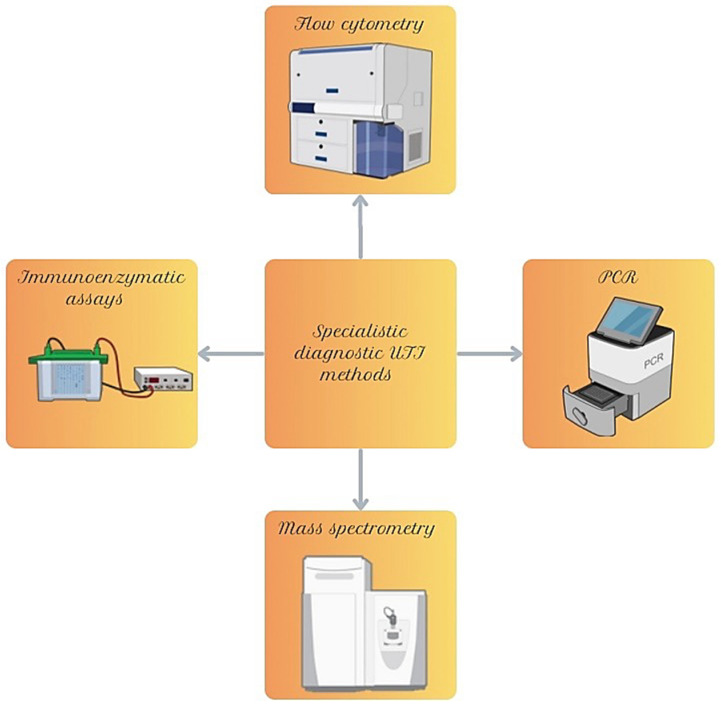
Specialized laboratory methods used in the diagnosis of urinary tract infections (UTIs).

Around the world, both hospital-based laboratories and specialized laboratories operate within healthcare systems. The former focus on routine microbiological diagnostics using basic techniques, such as cultures, microscopy, and standard methods of antimicrobial susceptibility testing, including the disk diffusion method. These laboratories are equipped to detect common pathogens and assess their resistance to antibiotics. Specialized laboratories, typically located within large academic centers and clinical hospitals, are equipped with advanced technological infrastructure that enables the identification of pathogens using molecular biology techniques and automated systems (Figure 3). These allow for rapid and precise detection of resistance genes, which is crucial in diagnosing infections caused by multidrug-resistant microorganisms. In addition to diagnostics, specialized laboratories often serve as reference centers, providing support in complex cases and engaging in research and educational activities. These laboratories are required to meet high-quality standards and undergo regular inspections.

### 4.1. Analysis of Selected Genes

The PCR method is widely known and used worldwide (Figure 3). It enables the production of multiple copies of a selected DNA fragment from a small sample of genetic material [88,89]. Real-time PCR is a more advanced method for analyzing small DNA fragments, characterized by the use of fluorogenic markers and fluorescence emission [90]. Both conventional PCR and real-time PCR are widely used worldwide and are characterized by high sensitivity and specificity. Their main advantage is ease of use—they require minimal manual intervention during the initial sample preparation, while all subsequent steps are carried out fully automatically, including the generation of test results. Ready-to-use reaction mixtures (kits) for multiplex real-time PCR, specific for multiple genes, are now available and designed to detect the most common carbapenemase genes encoding KPC, NDM, VIM, IMP, and OXA-48. Some kits also identify ESBL/AmpC genes [91,92]. Some systems allow direct detection of pathogens in samples collected from patients, which further shortens diagnostic time and improves efficiency.

GeneXpert (Cepheid, Sunnyvale, CA, USA) is one of the first and most widely used automated diagnostic systems worldwide, enabling the detection and identification of genes encoding the most common carbapenemases, such as KPC, NDM, VIM, IMP-1, and OXA-48 (including variants OXA-181 and OXA-232). One of the key advantages of this system is its ability to detect carbapenemase genes directly in stool samples, eliminating the need for bacterial strain isolation and culture. Moreover, the turnaround time for obtaining results is less than an hour, provided that the test is performed within a maximum of 6 h after sample collection. Studies have shown that GeneXpert exhibits high sensitivity and specificity, ranging from 84% to 100% [93,94].

Genie II^®^ (AmplexDiagnostics GmbH, München, Germany) is a diagnostic system based on LAMP (Loop-Mediated Isothermal Amplification) technology, which, unlike conventional PCR, does not require cyclic temperature changes, but instead maintains constant isothermal conditions. Each test consists of a set of interconnected tubes, containing lyophilized, ready-to-use reagents essential for the amplification of antibiotic resistance genes. The company offers several variants of the Eazyplex^®^ SuperBug tests, allowing the detection of the most common carbapenemase genes, including NDM, KPC, VIM, IMP, OXA-48, as well as their subtypes OXA-181 and OXA-232, and AmpC. The total testing time is approximately 15 min. The system enables real-time monitoring of amplification, with results displayed as amplification curves on the device screen and in tabular format. According to the manufacturer, Genie II^®^ tests can be used not only on bacterial cultures from solid media, but also directly on clinical samples obtained from patients, such as urine, rectal swabs and positive blood culture bottles. The system is characterized by high sensitivity (>92.3%) and specificity (100%), as confirmed by studies [95,96,97,98,99].

Another available automated diagnostic system is the BD MAX™ platform, which enables the detection of five major carbapenemase types: KPC, VIM, IMP, NDM, and OXA-48. The test can be performed both on bacterial cultures and directly on rectal swabs. The system is characterized by very high sensitivity and specificity, reaching 97.1% and 98.8% for bacterial cultures and 92.8% and 97.8% for rectal swabs. Due to its high accuracy, some studies suggest that the BD MAX™ platform could be used for screening purposes to detect carbapenemase-producing strains [100,101]. In some cases, the use of this system may lead to false-positive results. A study conducted by Young Yoo and colleagues, published in 2022, confirmed the high sensitivity, specificity, and negative predictive value (NPV) of the CheckPoints CPO test, but reported a low positive predictive value (PPV—60.8%), which was attributed to the frequent occurrence of false-positive results [102].

It is worth mentioning one of the newest automated diagnostic systems, the BioFire^®^ FilmArray^®^ System (bioMérieux), which allows for the identification of genes encoding carbapenemases, including NDM, KPC, VIM, IMP, and OXA-48, as well as ESBL genes from the CTX-M family. One of the key advantages of this system is its ability to perform direct analysis from a clinical sample, eliminating the need for prior bacterial culture, with results available in less than an hour. However, scientific literature has highlighted discrepancies between the results obtained using this system and those obtained through conventional culture-based methods. Researchers recommend caution when interpreting results, as the system does not always detect all genetic variants associated with a given resistance mechanism. It has been suggested that the presence of a genetic marker in a sample does not necessarily indicate that it corresponds to a specific pathogen, as it may originate from another bacterial strain present in the clinical material. Research findings indicate that, to accurately determine antimicrobial resistance, it is essential to perform bacterial culture and assess the antimicrobial susceptibility of the isolated strain [103,104,105,106].

### 4.2. Mass Spectrometry

One of the modern methods for microorganism identification is MALDI-TOF MS (matrix-assisted laser desorption/ionization time-of-flight mass spectrometry) (Figure 3). This technique is based on mass spectrometry, which enables the rapid and precise identification of bacteria. The analysis process begins with applying a bacterial colony onto a special plate and then mixing it with a chemical matrix, which facilitates ionization. The sample is then irradiated with a laser beam, leading to the release of cellular proteins. The resulting protein profile is analyzed in a mass spectrometer [107]. This technology is used, among others, for the detection of carbapenemases (KPC, NDM, VIM, IMP, OXA-48) [108] or cephalosporinases (ESBL, AmpC) [109], colistin resistance mechanisms (MCR) [110] or MRSA [111]. It is emphasized that this system can be an effective tool for the rapid identification of bacteria and their resistance mechanisms; however, its reliability depends on the standardization of culture conditions, including the use of the same media and incubation times [112]. Additionally, it is suggested that combining MALDI-TOF MS with machine learning algorithms allows for the instant identification of bacterial strains, enabling resistance diagnosis within just a few minutes. This method proved to be an effective method for the rapid detection of beta-lactamase activity, showing high concordance with PCR results. It can provide reliable results within just 2 h, making it a valuable tool in clinical diagnostics [113,114,115,116].

### 4.3. Immunoenzymatic Assays

The ELISA (Enzyme-Linked Immunosorbent Assay) test is an immunoenzymatic method used for detecting antigens or antibodies based on the antigen-antibody reaction (Figure 3). The procedure involves coating a microplate with antigens or antibodies, adding a sample, and then introducing enzyme-labeled antibodies, which bind to the target antigen. After adding the substrate, the enzyme catalyzes a colorimetric reaction, and the intensity of the color change is measured spectrophotometrically, allowing for the quantitative determination of the detected substance [117]. The test is used to detect β-lactamases, including ESBL (CTX-M, TEM, SHV), AmpC (CMY, DHA), and carbapenemases (KPC, NDM, VIM, IMP, OXA-48) through the presence of enzymes that hydrolyze β-lactam antibiotics. The results of the study showed that the ELISA-KPC test demonstrated 100% sensitivity and 89% specificity in detecting KPC enzymes, including their variants [118]. Additionally, this study demonstrated that ELISA tests developed with polyclonal rabbit antibodies against SHV-1 and CMY-2 beta-lactamases were highly specific and sensitive, achieving at least 95% sensitivity and specificity in detecting these enzymes. The ELISA assays showed no cross-reactivity with unrelated beta-lactamases and were able to detect picogram quantities of purified proteins, with detection limits correlating to sequence homology levels [119].

### 4.4. Flow Cytometry

Flow cytometry is an advanced analytical technique that enables rapid and multiparametric analysis of individual cells in suspension (Figure 3). This method involves passing cells through laser beams, allowing for the assessment of their properties based on light scattering and fluorescence. The light signals are recorded by detectors, converted into digital data, and analyzed by a computer [120]. Studies have shown that urinary flow cytometry can serve as an alternative to traditional bacterial culture, which is time-consuming and costly, as well as to dipstick tests, which often produce results with low sensitivity and specificity. In a study involving 281 patients, it was demonstrated that if the bacterial count in urine was below 60 bacteria per microliter, a urinary tract infection could be reliably excluded. As a result, nearly half of the urine cultures would have been unnecessary, and the number of patients receiving unwarranted antibiotic treatment could be significantly reduced [121,122,123]. Moreover, urine analysis using flow cytometry with the Sysmex UF-1000i system has been recognized as an efficient and rapid method for detecting UTIs, compared to dipstick tests and microscopic sediment analysis. Bacterial count has been identified as the most sensitive and specific marker of UTI in symptomatic patients [124]. Additionally, it has been suggested that in the future, combining flow cytometry with MALDI-TOF MS technology could allow for the direct identification of bacteria in positive urine samples, leading to faster and more precise antibiotic selection. Currently, flow cytometry is primarily used to detect and quantify bacteria and other urine components, but it does not allow for the identification of resistance mechanisms or the precise determination of whether bacteria are Gram-positive or Gram-negative. For comprehensive diagnostics and effective treatment selection, additional tests, such as culture, PCR, or MALDI-TOF MS, are required [122].

## 5. Modern Technologies and Future Directions in UTI Diagnostics

**Figure 4:**
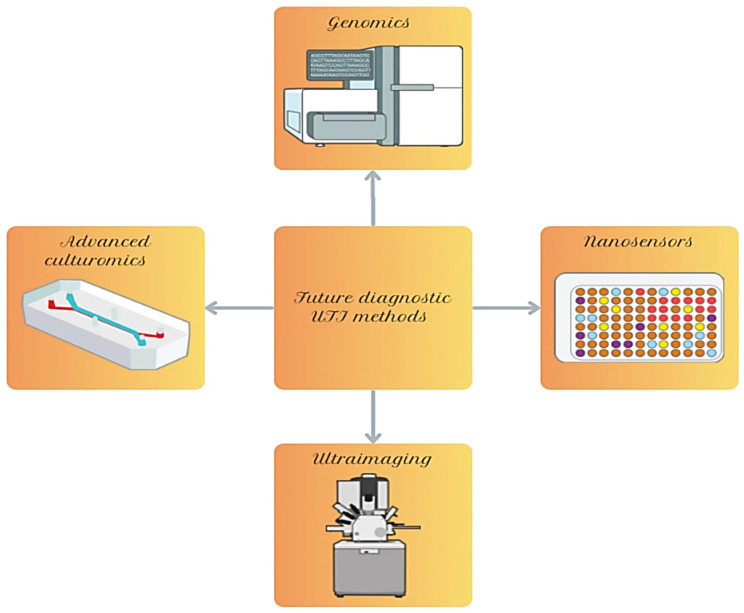
Laboratory methods with the potential to be introduced in the future for the diagnosis of urinary tract infections (UTIs).

As mentioned, UTIs represent a growing global problem. In order to effectively prevent, detect, and treat them, the application of the latest research techniques and the improvement of existing ones may enhance UTI diagnostics, potentially enabling more sensitive, specific, or faster results in the future (Figure 4). The ultimate clinical impact of the diagnostic tools described below will depend on the method used, result interpretation, costs, regulatory status, the population tested, as well as the rapidly expanding knowledge in the field of UTI microbiology [23,33].

### 5.1. Advanced Methods of Genomics and Genetic Modifications

Metagenomics is a method that involves direct sequencing of genetic material obtained from a urine sample, without the need for culturing pathogenic microorganisms (Figure 4). This method uses hybridization probes to capture specific DNA sequences of pathogens. Unlike PCR, metagenomics enables simultaneous screening of hundreds of microorganisms in a single analysis. Although it still relies on known sequences, it offers a much broader view than traditional targeted tests. For this reason, the authors of the discussed study concluded that metagenomics may, in the future, replace commonly used diagnostic methods such as PCR [125]. The results showed 100% positive predictive agreement with culture results, which identified only 13 different microorganisms—compared to 19 detected by PCR and as many as 62 detected using precision metagenomics. The precision metagenomics enables simultaneous quantitative analysis and phenotypic classification of microorganisms using bioinformatic platforms such as Explify^®^, which in practice provides detailed data necessary for rapid and accurate empirical treatment of UTIs [126]. Moreover, the authors of the study demonstrated that metagenomics showed significantly higher diagnostic effectiveness in determining the etiology of infections compared to urine culture in kidney transplant recipients with recurrent UTIs, as antimicrobial therapy was modified in only two cases (33.3%) based on culture results, thanks to metagenomics, in as many as 12 cases (76.9%). The study was conducted in a population of adult kidney transplant recipients with documented recurrent UTIs, who, due to immunosuppression, are particularly susceptible to infections with complex etiologies. The material analyzed consisted of urine samples collected during episodes of symptomatic UTI. In this clinical group, conventional urine culture often demonstrates low sensitivity, especially in cases of polymicrobial infections or infections caused by fastidious organisms [127]. In another article, studies involving hospitalized patients with UTIs that compared metagenomic next-generation sequencing (mNGS) to standard urine culture were included. The clinical outcomes evaluated included laboratory turnaround time and antibiotic use. It was demonstrated that mNGS showed a significantly higher ability to detect polymicrobial infections, and the laboratory turnaround time appeared shorter compared to standard culture [128].

Whole genome sequencing (WGS) is a technique that involves reading the complete DNA sequence of a given pathogen. DNA is obtained directly from the sample, fragmented into small pieces, which are then sequenced, and the resulting reads are assembled into a complete genome using specialized software [129]. A very interesting case that highlights the important role WGS may play in the future is the clinical report described below. The study found that the isolated *E. coli* strain possessed diverse antibiotic resistance genes, confirmed by both WGS and phenotypic resistance testing. It was concluded that WGS plays a key role in monitoring and preventing the growing threat of antimicrobial resistance [130]. The focus was on assessing genetic diversity, the presence of antibiotic resistance genes, virulence genes, and mobile genetic elements that may contribute to the spread of resistance, which further emphasizes the usefulness of the described method.

Another noteworthy diagnostic tool is the CRISPR-Cas system, which enables the recognition of specific DNA fragments in samples, such as urine. In this method, a Cas protein is used, whose task is to identify genetic material characteristic of a given pathogen. Upon detection, the protein is activated and cuts specially added molecules—their cleavage causes light emission or a color change, which indicates a positive result [131]. The significant potential of this technology was confirmed in a study from 2017. Researchers used the CRISPR-Cas9 system to precisely delete *luxS* in the *E. coli* SE15 strain—one of the main pathogens responsible for UTIs. The *luxS* is responsible for the production of a signaling molecule (AI-2), which participates in bacterial communication and biofilm formation. *E. coli* mutants lacking *luxS* showed a significantly reduced ability to form biofilm compared to the wild-type strain [132]. In another study on the CRISPR-Cas system, it was stated that it is a new generation of gene editing technology, and CRISPR-based diagnostics are easy to use, easy to carry, and take less time than the real-time PCR-based assays currently in use [133].

### 5.2. Nanosensors

Nanotechnology offers great potential in the development of rapid diagnostic methods characterized by high sensitivity, specificity, and low cost, enabling the detection of infections caused by various pathogens (Figure 4) [47]. The length of these DNA strands influences their stronger interaction with the gold surface and the greater force required to detach them, which directly affects the light emission by the attached fluorophores. Each bacterial species causes a characteristic change in light emission [134].

An example of such sensors includes Ami-AuNPs-DNA sensors (based on gold nanoparticles modified with amino groups and DNA). They enable effective differentiation of the five main pathogenic bacteria of the urinary tract based on their unique “fingerprint” signal patterns, and demonstrate sufficient sensitivity to detect single bacteria with a detection limit of up to 10^7^ colony-forming units per milliliter (CFU/mL). Moreover, the sensor has been successfully used to identify bacteria in urine samples from patients with clinically confirmed UTIs. Based on this, researchers concluded that the developed fluorescent sensor can be used for rapid and accurate discrimination of bacterial pathogens of the urinary tract, serving as a promising diagnostic tool for detecting diseases caused by bacterial infections [135].

Another biosensor that effectively detects *E. coli* is the UCNP-Apt/GO@Fe_3_O_4_ sensor (upconversion nanoparticles conjugated with an aptamer/graphene oxide decorated with magnetite, Fe_3_O_4_). This fluorescent biosensor is based on the Förster Resonance Energy Transfer (FRET) mechanism, a physical phenomenon in which energy from one fluorescent molecule, the donor, is directly transferred to another molecule, the acceptor, without photon emission, provided both are in very close proximity. UCNP nanoparticles act as fluorescence donors, while GO@Fe_3_O_4_ (a nanocomposite of graphene oxide and iron oxide) exhibits both adsorptive and fluorescence-quenching properties. A single-stranded DNA molecule (aptamer) that specifically recognizes *E. coli* is attached to the surface of the UCNP. Initially, when UCNP-Apt is bound to GO@Fe_3_O_4_, fluorescence is quenched. In the presence of *E. coli*, the aptamer recognizes the bacteria and detaches from GO@Fe_3_O_4_, resulting in the recovery of fluorescence. The signal intensity is proportional to the bacteria concentration, allowing for precise detection. This sensor features high sensitivity (467 CFU/mL) and a short analysis time (30 min). However, researchers note that the potential for fully using this biosensor in infection diagnostics requires further optimization of the detection limit [136].

### 5.3. Ultraimaging

Scanning Electron Microscopy (SEM) is an advanced imaging technique that uses a beam of electrons to obtain detailed, three-dimensional images of the surface of examined samples. In the diagnosis of UTIs, SEM allows for the direct visualization of microorganisms and their morphology. A limitation of this method is the need for specialized sample preparation [137]. Additionally, this method is not capable of determining the genus or species of the microorganism being analyzed [138]. Attention should also be given to a study describing the use of SEM and Energy Dispersive X-ray (EDX) analysis. These methods are frequently used in scientific research, but are not routinely applied in hospital laboratories. In the mentioned study, researchers analyzed 206 urine sediment samples in search of bacteria, crystals, epithelial cells, as well as white and red blood cells. It was suggested that this method could be used for the detection and identification of microorganisms and crystals present in urine samples—after all, urolithiasis is one of the many factors that contribute to UTIs. SEM enables a broad overview of the sample, allowing for a general description of its composition and the preliminary identification of microorganisms and cells, while EDX analysis reveals the elemental composition of the crystals. These two complementary techniques may serve as useful tools in supporting urine analysis [139].

### 5.4. Modern Methods of Culturing Microorganisms and Their Biofilms

Methods for culturing microorganisms responsible for UTIs have evolved over the years. Currently, previously described selective and chromogenic media are used; however, there is still a search for methods that more accurately reflect the natural conditions in which UTI-causing bacteria multiply (Figure 4).

One of the modern techniques is the use of artificial urine. Although research on its composition has been ongoing for over 20 years [140], only in recent years has it been refined enough to faithfully replicate the actual composition of human urine. The main challenge is the high variability in the chemical composition of human urine. The study discussed below presents the composition of such a preparation. Liquid chromatography coupled with mass spectrometry (LC-MS) was used. In this study, the researchers also decided to remove iron from the formulation. It was shown that gene expression profiles of UPEC strains grown in artificial urine and PHU (pooled human urine) were similar—fewer than 7% of genes showed differences in expression between the two conditions [141]. If the conditions differed significantly, a larger number of genes would have been activated to help the bacteria adapt to the new environment. Such a small difference in gene expression clearly indicates a high degree of similarity between the composition of the artificial preparation and natural urine. In another study, a synthetic human urine medium (Composite SHU medium) was developed as an artificial model of human urine that supports the growth of both Gram-positive and Gram-negative bacteria associated with UTIs. The study showed that Gram-negative bacteria grew well even without additives, while Gram-positive bacteria required supplementation with yeast extract (at least 0.2%). Bacterial growth was also improved by adjusting culture conditions, such as increasing the culture volume and using shaking incubation. According to the researchers, this medium is inexpensive, easy to prepare, chemically stable, and closely mimics the composition of natural urine, making it a valuable tool for studying UTIs. The authors emphasize that this medium can help standardize microbiological research on bacteriuria and support the development of new diagnostic and therapeutic methods [142].

In one study, it was shown that the ability to form biofilm was strongly correlated with antibiotic resistance, especially in the case of ESBL and MDR *E. coli* strains [143]. Currently, specialized machines are used to study biofilm formation and bacterial growth under controlled conditions. One of the most commonly used devices is the CDC Biofilm Reactor^®^. In one study, researchers examined whether the position and orientation of the surfaces (so-called coupons) within the reactor influenced the amount of biofilm formed. Biofilms of *P. aeruginosa* and *S. aureus* were analyzed. For *P. aeruginosa*, no differences were observed—the biofilm grew equally intensively on every surface, regardless of its position in the reactor. In the case of *S. aureus*, no statistically significant differences were found, but the results were more variable, which made it impossible to confirm that all positions had the same influence on growth. Additionally, it was observed that the structure of the biofilm differed depending on the reactor side—biofilm on the side exposed to higher shear forces had a different structure than on the quieter, glass side. Although position did not significantly affect the biofilm quantity, the authors suggested that these structural differences might influence antibiotic sensitivity and warrant further research [144]. A new type of reactor—with glass beads—was recently developed, enabling biofilm growth with a small volume of broth. This allows for large-scale studies, from simple laboratory tests to preclinical models. In a comparative study, it was shown that *S. aureus* biofilm grown on beads was more susceptible to ertapenem and tobramycin than biofilm grown on coupons. For *P. aeruginosa*, the differences in treatment response were smaller, but a greater reduction in bacterial count was still observed on the beads after tobramycin treatment. These results suggest that the glass bead reactor may be an effective tool for testing antibiotic efficacy and translating results from in vitro models to in vivo systems, supporting the development of new antimicrobial technologies [145].

## 6. Limitations and Future Perspectives of UTI Diagnostics

It should be borne in mind that each diagnostic method has its advantages and limitations (Table 4). The latest technological innovations may offer many benefits; however, they still face different important limitations, and therefore, urine culture remains the gold standard for UTI diagnostics [146]. Modern microbiology laboratories are increasingly using automated diagnostic systems, including VITEK 2 and BD Phoenix, which allow for fast and precise identification of microorganisms and assessment of their antibiotic susceptibility. An alternative to these is genetic techniques, such as PCR and fully automated systems based on real-time PCR, which offer enormous diagnostic potential, but, for now, require cautious interpretation of results and complementary culture-based testing. It is important to consider the clinical implications of these methods. Techniques such as metagenomics or NGS can also detect DNA from non-pathogenic microorganisms, which may complicate the interpretation of results. Additionally, most of these diagnostic approaches require over an hour to deliver results. As a consequence, patients often receive empirical antibiotic therapy first, which is only later adjusted to a targeted regimen once definitive results are available [147,148]. Although the per-test costs are not particularly high, the expense of the necessary equipment and the need for specialized laboratory personnel significantly limit the widespread implementation of these methods [128,149,150]. It is also important to consider the possibility of false-negative results and sample contamination. Despite its high specificity, MALDI-TOF MS, similar to other direct identification methods, may detect bacteria representing colonization or resulting from sample contamination [151,152]. Moreover, current MALDI-TOF MS data analysis software is not capable of reliably identifying all microorganisms present in mixed microbial populations [153,154]. Consequently, contaminated urine samples often yield inconclusive or clinically insignificant results. In the case of NGS, co-infections are common, with mixed bacterial–viral infections being the most frequently observed [155]. Among modern techniques in development, attention has been drawn to novel molecular approaches (metagenomics and WGS), ultraimaging with SEM, and fluorescent nanosensors. All of them provide promising results in detecting the most common uropathogens, although further optimization of the detection threshold is needed. As an alternative to this, the use of artificial urine as a culture medium for the growth and biofilm development of uropathogens appears to be another approach to study the physiology of these microorganisms by mimicking the conditions of the urinary tract.

Despite technological progress, many scientists and clinicians emphasize the need for further research in this area and the combination of different methods to obtain a more comprehensive diagnostic picture and to ensure effective treatment selection. In addition to clinical and analytical factors, new diagnostic tests must be evaluated within a broader context of the healthcare system. The potential clinical value of new technologies is always considered alongside their financial implications—for the patient, the provider, the payer, and the laboratory alike [152]. The need to establish both analytical and clinical validation, including assessment of concordance, repeatability, and limits of detection, is emphasized. Another challenge is the lack of standardization between different platforms and laboratories, which may lead to variability in quality and comparability of results. An important aspect is also the way results are reported—current reporting formats often do not account for clinical context, which may create a risk of overdiagnosis or overtreatment, especially in cases of colonization or detection of microbiota with unclear pathogenic significance [156]. The implementation of new diagnostic technologies into clinical practice requires initiation of pilot studies and evaluation of their clinical utility, taking into account the impact of these methods on therapeutic decision-making, time to initiation of treatment, and patient health outcomes. The development of standards for result interpretation and clinical management algorithms will ensure safe and rational use of new tests [157].

In Poland, the diagnosis of UTIs is primarily made based on urine culture with a quantitative assessment and urinalysis, including the evaluation of leukocyturia and the presence of nitrites. Clinical symptoms and patient predispositions to UTIs (such as urolithiasis or the presence of a urinary catheter) also play a crucial role. In local hospital microbiology laboratories, cultures are most commonly performed using Columbia agar with 5% sheep blood, MacConkey agar, and Sabouraud agar. Species identification of bacteria and yeasts is carried out mostly with the use of the VITEK 2 Compact system, and reference strains for quality control. Susceptibility assessment is conducted mostly by the disk diffusion method, for instance, on Mueller–Hinton agar for Gram-negative rods, staphylococci, and enterococci, and on Mueller–Hinton agar supplemented with 5% defibrinated horse blood and 20 mg/L NAD for streptococci. If needed, minimum inhibitory concentrations are determined using antibiotic gradient strips or the VITEK 2 Compact system [34]. In higher-reference laboratories, MALDI-TOF MS, PCR-based assays, fluorescent flow cytometry analyzers, as well as automated urine analysis systems based on digital imaging with automatic recognition of urinary sediment particles, are commonly applied [158,159].

## 7. Conclusions

UTIs are a significant global health problem, and therefore an effective prevention, proper diagnostics, and appropriate treatment are key to improving outcomes in this area of medicine. This review focuses on current trends and modern diagnostic solutions for detecting uropathogens, which may become the standard in everyday clinical practice in the future. Based on the literature review, the following conclusions can be drawn:

- Culture methods remain the gold standard for UTI diagnosis;
- Automatic analysis systems based on spectrophotometric measurements or microbial genetic/protein profiles are gaining importance, but are limited by price and staff training;
- The newest methods, such as nanosensors or cultivation of biofilms in microfluidic systems, are very promising, although, for now, are poorly validated.

## Figures and Tables

**Table 1:** Risk factors of complicated urinary tract infections (UTIs) with examples [23,28].

Risk Factor	Examples
Anatomical abnormalities	Bladder diverticula, posterior urethral valve
Foreign body	Catheter, stent, nephrostomy
Obstruction	Enlarged prostate, stones, kidney cysts
Functional disorders	Vesicoureteral reflux, neurogenic bladder
Other	Pregnancy, diabetes, immunosuppression, resistant pathogens

**Table 4:** Characteristics of laboratory methods used in the diagnosis of urinary tract infections (UTIs).

Methods [Reference]		Time	Sensitivity	Resistance Detection	Costs	Bedside Test	Limitations
Classical molecular methods	Genie expert [93,94]	<1 day	+++	Detects only known and defined resistance mechanisms (mainly carbapenemases)	+++	+/−	No distinction between colonization and infection Limited by its inability to detect all resistance determinants, particularly those outside the targeted panel
	Genie II [95,96,97,98,99]	<15 min		Detects only known and defined resistance mechanisms (mainly ESBL and carbapenemases)	++	+	
	BD-MAX [100,101,102]	2–2.5 h			++	−	
	Bio-Fire [103,104,105,106]	>1 h			+++		
MALDI-TOF MS [107,110,111,112,113,114,115,116]		>2 h	+++	Detects cephalosporinases, carbapenemases, MCR, MRSA, VRE	+	−	No distinction between colonization and infection Differences with urine culture results
ELISA [118,119]		1.5–2.5 h	++	Enables direct prediction of beta-lactam resistance	+	−	Risk of cross-reactions Detects mechanism of resistance, but not a microbial strain
Flow cytometry [120,122,124]		3–10 min	+	−	++	−	Leukocyturia or biofilm may distort the result
Advanced molecular methods	Metagenomics [126,127]	24–48 h	++++	Can analyze hundreds of resistance genes	++++	−	Time-consuming No distinction between colonization and infection
	Whole genome sequencing [129,130]	24–72 h		Can analyze all resistance genes		−	Time-consuming No distinction between colonization and infection
	CRISP [131,132,133]	30–60 min		Can analyze specific resistance genes	++	+/−	Risk of false positive results Limited numbers of sets available on the market
Nanosensors [47,134,135,136]		10–30 min	+++	−	++	−	The need for additional tests Limited number of sets available on the market
SEM [137]		<24 h	+	−	+++	−	Limited to visualization of microorganisms, but unable to indicate a microbial strain and the mechanism of resistance
Culture	Classical media [61,62,63]	>24 h	+	Detects cephalosporinases, carbapenemases, MCR, MRSA, VRE	+	−	Time-consuming and requiring a set of disposable materials to detect different resistance mechanisms
	Artificial urine [141,142,144,145]	>24 h		Theoretically able to detect mechanisms of resistance (unvalidated)	+		Time-consuming and requiring a set of disposable materials to detect different resistance mechanisms Unvalidated

Legend for Sensitivity: + low, ++ moderate, +++ high, ++++ very high; Legend for Costs: + very low cost (≤8 USD), ++ moderate cost (8–27 USD), +++ high cost (27–135 USD), ++++ very high cost (>135 USD); Legend for Bedside test: + possible; − impossible; +/− not classic bedside tests; however, due to their cartridge-based format and high level of automation, they can be used as near-patient diagnostics.

## Data Availability

No new data were created or analyzed in this study. Data sharing is not applicable to this article.

## Supplementary Materials

The following supporting information can be downloaded at: https://www.mdpi.com/article/10.3390/ijms262210847/s1.
