# Supplementary figures and images for: A Review on the Current and Future State of Urinary Tract Infection Diagnostics
Source: Int J Mol Sci. 2025 Nov 8;26(22):10847. doi: 10.3390/ijms262210847 (PMC12652086; doi:10.3390/ijms262210847)

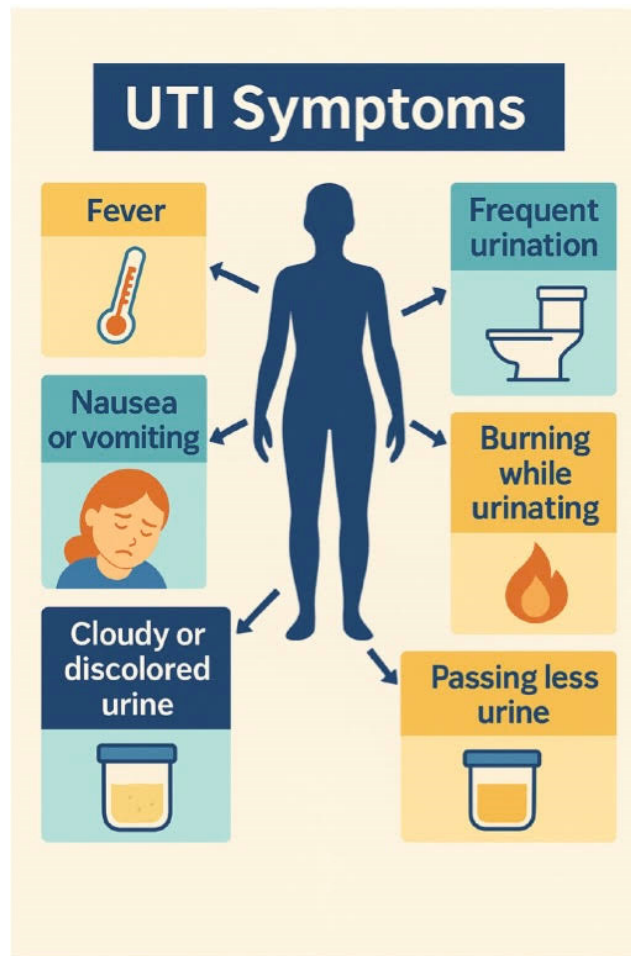

**Figure S1.** Typical symptoms presented during urinary tract infections (UTIs).

Supplement: Supplementary file 1 [file ijms-26-10847-s001.zip › ijms-3944178-supplementary.pdf]
